# Axillary metastasis from occult breast cancer and synchronous contralateral breast cancer initially suspected to be cancer with contralateral axillary metastasis: a case report

**DOI:** 10.1186/s12905-021-01569-x

**Published:** 2021-12-17

**Authors:** Myung Won Song, So Yeon Ki, Hyo Soon Lim, Hyo-jae Lee, Ji Shin Lee, Jung Han Yoon

**Affiliations:** 1 Department of Radiology, Chonnam National University Hwasun Hospital, Chonnam National University Medical School, 322 Seoyang-ro, Hwasun-eup, Hwasun, 58128 Korea; 2 Department of Pathology, Chonnam National University Hwasun Hospital, Chonnam National University Medical School, Hwasun, Korea; 3 Department of Surgery, Chonnam National University Hwasun Hospital, Chonnam National University Medical School, Hwasun, Korea

**Keywords:** Axilla, Breast neoplasms, Lymphatic metastasis, Diagnosis, Case report

## Abstract

**Background:**

Initial detection of axillary metastasis without known ipsilateral breast cancer could be a challenging diagnostic problem. Four options could be considered for the primary site of the malignancy: ipsilateral occult breast cancer, contralateral breast cancer, tumors in other distant organs, and primary axillary malignancy itself. Although breast cancer is known as the most common primary cancer of axillary metastasis, both occult breast cancer and breast cancer with contralateral axillary metastasis (CAM) are rare.

**Case presentation:**

A 63-year-old woman presented with palpable right axillary metastasis, and a tiny contralateral breast cancer was detected by breast magnetic resonance imaging. No lesion was found in the ipsilateral right breast and contralateral left axillary region. Both right axillary metastasis and contralateral breast cancer were positive for estrogen receptor. The diagnostic issue was to determine whether the axillary metastasis was derived from the contralateral breast cancer or not. Right axillary dissection and left breast conserving surgery were performed. The final diagnosis was occult breast cancer that presented with axillary lymph node metastasis and early-stage synchronous contralateral breast cancer, based on clinical evidence and postoperative pathologic results. After surgery, systemic treatment and whole breast irradiation were administered. No recurrence or metastasis was observed 15 months postoperatively.

**Conclusion:**

For accurate diagnosis of axillary metastasis without detectable ipsilateral breast cancer, multifaceted diagnostic approach considering clinical, radiological, and pathological evidences is required.

## Background

Axillary metastasis detected in patients without a known history of primary malignancy is mostly found to originate from breast cancer [1]. However, in the absence of any evidence of breast cancer on physical examination and regular imaging studies, diagnosis of primary cancer might be quite challenging. In those cases, more advanced investigation is required, such as thorough review of breast examination findings, breast magnetic resonance imaging (MRI), pathological analysis of percutaneous biopsy specimens, and additional whole-body imaging studies for identifying primary malignancies other than breast cancer. Sometimes, clinically occult breast cancer may be finally detected by breast MRI or pathological analysis of mastectomy specimen, which has not been apparent on physical examination, mammography, and ultrasonography (US) [1, 2]. However, primary breast cancer might remain undetected despite the pathologic features of the metastatic lymph node suggesting its origin from the breast.

Herein, we reported a case of occult breast cancer presenting with axillary metastasis and synchronous contralateral early-stage breast cancer; such cancers might be misdiagnosed as breast cancer with contralateral axillary metastasis (CAM).

## Case presentation

**Fig. 1:**
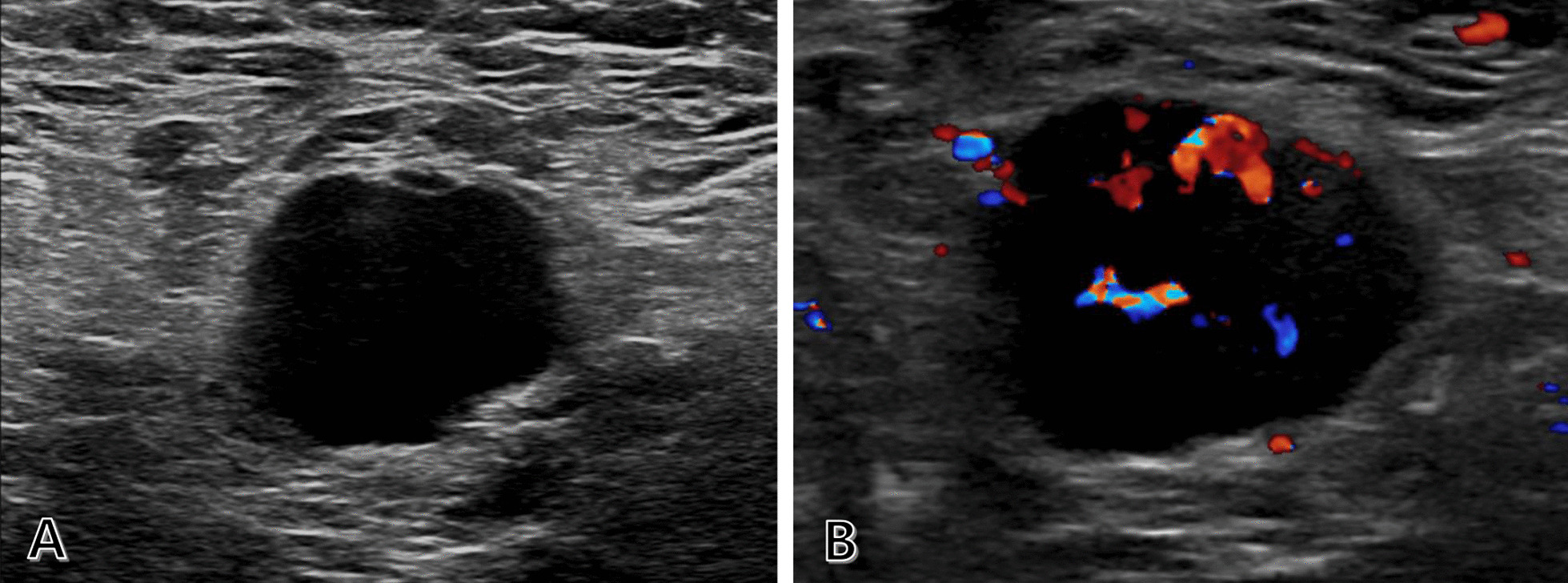
Metastatic lymph node in the right axilla that presented as a palpable mass. **A** Ultrasound image showing an irregular mass with marked hypoechogenicity in the right axilla. **B** Doppler ultrasound image showing increased vascularity in the lesion

**Fig. 2:**
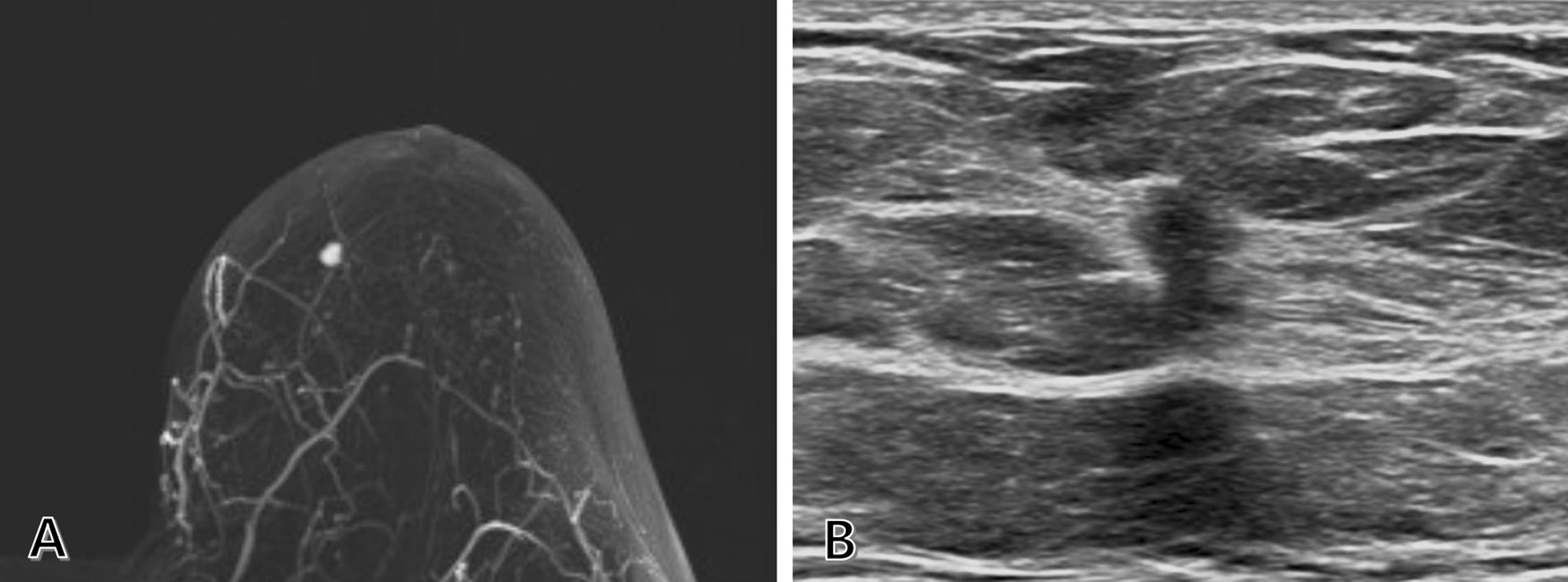
Early-stage left breast cancer. **A** MRI revealing a 0.6-cm enhancing mass in the left upper inner breast, on the contralateral side of the metastatic axillary lymph node. **B** Second-look ultrasound showing a 0.6-cm irregular, indistinct, isoechoic lesion, corresponding to the mass detected by MRI. MRI, magnetic resonance imaging

A 63-year-old woman visited a clinic for a palpable right axillary mass. US showed a 2.5-cm irregular hypoechoic mass in the right axilla, which was considered to be a malignant lymph node such as metastasis (Fig. 1). Similar to physical examination and mammography, US revealed no remarkable findings in both the breasts and the left axillary region. A 14-gauge core-needle biopsy revealed the right axillary lymph node as a metastatic carcinoma, possibly from the breast, with positive estrogen receptor (ER) status. Breast MRI was performed to determine the presence of occult breast malignancy. No lesions were detected in the right breast, whereas a 0.6-cm irregular enhancing mass was observed in the left upper inner breast, assessed as breast imaging reporting and data system (BI-RADS) category 4C (Fig. 2A). To exclude primary malignancies other than breast cancer as the origin of the metastasis, chest computed tomography (CT) and whole-body positron emission tomography-computed tomography (PET-CT) were performed. They revealed no specific findings, except the known right axillary metastasis.

Second-look US showed a 0.6-cm irregular, indistinct, isoechoic mass in the left upper inner breast, corresponding to the mass observed on breast MRI (Fig. 2B). US-guided 14-gauge core-needle biopsy confirmed the mass as an invasive carcinoma of no special type. The ER status of the cancer was positive, same as that of right axillary metastasis. Therefore, the right axillary metastasis was suspected to originate from the left breast cancer, and surgery was planned for its management and to confirm the diagnosis.

**Fig. 3:**
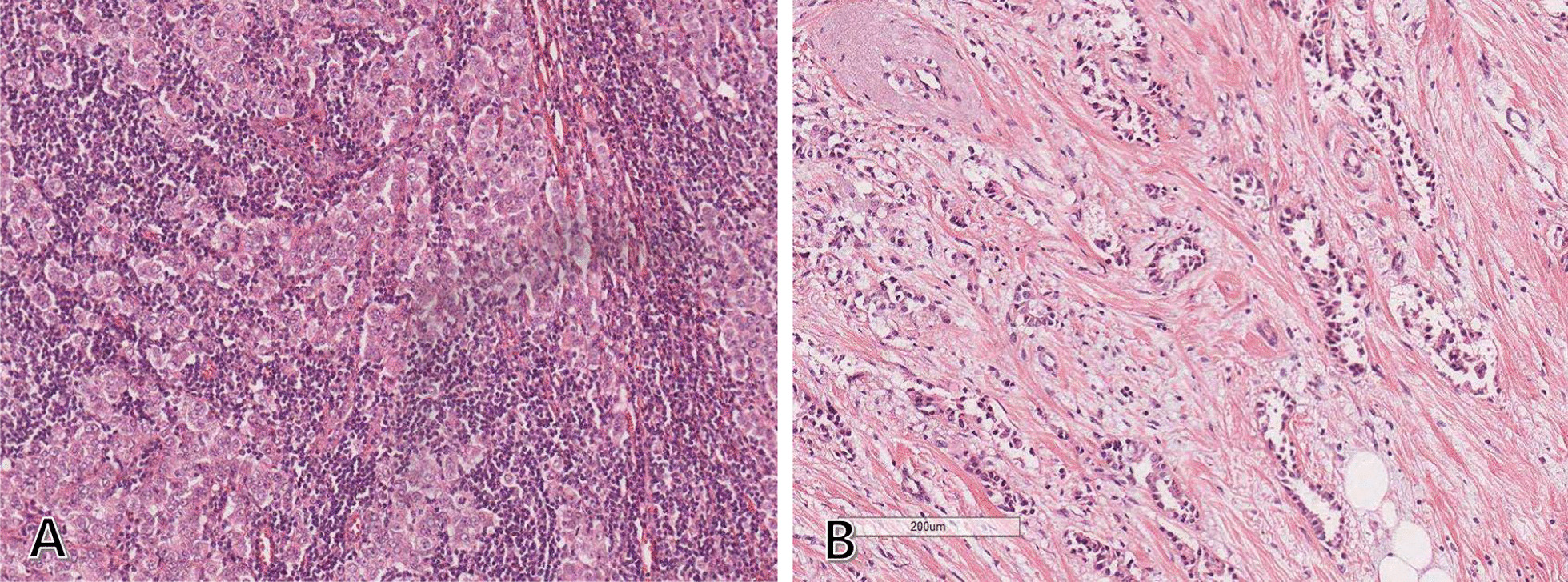
Histologic differences between the metastatic right axillary lymph node and left breast cancer. **A** Metastatic tissue from the right axillary lymph node showing poorly formed glands and nests of atypical cells, suggesting high-grade carcinoma (H&E stain, 100 × magnification). **B** Left breast cancer specimen showing well-differentiated glands, cells with fairly uniform nuclei, and well-preserved stromal tissue, suggesting low-grade carcinoma (H&E stain, 100 × magnification). H&E, Hematoxylin and eosin

**Fig. 4:**
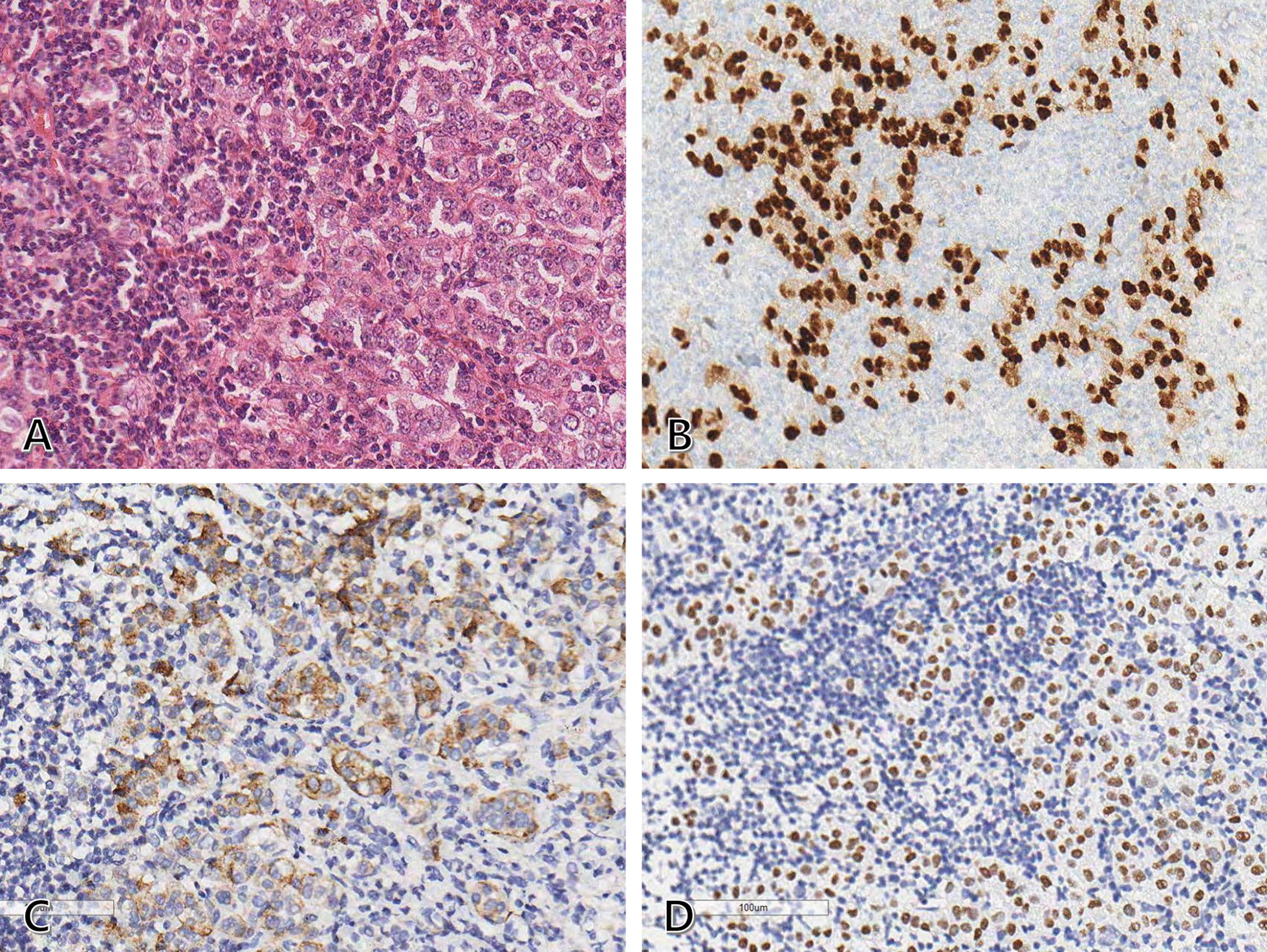
Histopathologic findings of the right axillary lymph node metastasis. **A** Hematoxylin and eosin staining showing a poorly differentiated metastatic tumor with nuclear pleomorphism and high mitotic activity (200 × magnification). **B**–**D** Immunohistochemical staining revealing that the tumor cells are positive for ER, GCDFP-15, and GATA3 (200 × magnification). These findings suggest that the metastasis is derived from the breast carcinoma. ER, Estrogen receptor; GCDFP-15, gross cystic disease fluid protein-15; GATA3, GATA binding protein 3

Right axillary lymph node dissection and left breast conserving surgery with sentinel lymph node biopsy were performed. The invasive carcinoma in the left breast was 6 mm in size, with cells positive for ER and progesterone receptor (PR) and negative for human epidermal growth factor receptor 2 (HER2). It was a low-grade invasive cancer with low Ki-67 expression (5%), histologic grade 1, and without lymphovascular invasion. Sentinel lymph node biopsy confirmed no left axillary involvement. In the right axilla, three macrometastatic lymph nodes were discovered, including the known palpable metastatic lymph node. Histologic findings of cancer cells in all three lymph nodes were fairly different from those of cancer cells in the left breast (Fig. 3). Examination of the right axillary metastasis showed high-grade carcinoma with poorly formed glands and nests of atypical cells, while examination of the left breast mass showed low-grade cancer with well-differentiated glands and fairly uniform nuclei. These findings clearly indicated that the metastasis to the right axillary lymph node did not originate from the left breast cancer, although both of them showed ER positivity. Metastatic cancer cells in the right axilla were also positive for gross cystic disease fluid protein-15 (GCDFP-15) and GATA binding protein 3 (GATA3), suggesting that the metastasis was derived from the breast (Fig. 4).

Finally, the case was diagnosed as bilateral breast cancer consisting of occult right breast cancer with axillary metastasis (TxN1M0) and early-stage left breast cancer (T1bN0M0). Four cycles of doxorubicin and cyclophosphamide followed by four cycles of taxane (AC-T) chemotherapy, radiation therapy for each breast, and hormone therapy were administered after surgery. No recurrence or metastasis was observed 14 months postoperatively.

## Discussion and conclusions

When a case of malignant axillary lymph nodes without evidence of ipsilateral breast cancer is encountered, four options could be broadly considered for the primary site of the malignancy: ipsilateral occult breast cancer, contralateral breast cancer, tumors in other distant organs, and primary axillary malignancy itself. To identify the tissue of origin for the axillary lymph node metastasis, rigorous evaluation involving sufficient imaging workup is required. Breast MRI is essential for the evaluation of breast cancer with high sensitivity, given that it has been shown to diagnose more than two-thirds of breast cancer cases that were considered to be occult disease on physical examination, mammography, and US [3]. Furthermore, lymph node biopsy with pathologic examination helps in identifying the organ of primary cancer, including the breast. Immunohistochemical staining of specific markers, such as carcinoembryonic antigen, cytokeratin 7/20, ER, PR, and GCDFP-15, plays an important role in this investigation.

In our case, MRI revealed contralateral breast cancer, which was not detected by mammography and the first US. There were two possibilities: a breast cancer with synchronous CAM, and axillary metastasis from occult breast cancer with contralateral early breast cancer. Both CAM and occult breast cancer are not common; the incidence of CAM has been reported to be 1.9%-6% [4, 5], and occult breast cancer accounts for up to approximately 1% of breast cancers [6–8]. Additionally, axillary metastasis from an occult cancer would make the final diagnosis of bilateral synchronous breast cancer in our case, which is also rare with reported incidence of 0.2–3% of newly diagnosed breast cancers [9].

Although postoperative pathologic report was decisive for diagnosis, some clinical and pathological clues also indicated that our case was not consistent with CAM. In the literature, most breast cancers with synchronous CAM were found to be locally advanced cancers, and almost 90% of them were stage III carcinomas. They were all accompanied by ipsilateral metastatic lymph nodes (mostly N2 and N3) [5, 10]. Additionally, in most cases, breast cancer with CAM shows aggressive biologic characteristics such as higher tumor grade, frequent lymphovascular invasion, larger primary tumors, hormone receptor negativity, and HER2 overexpression [5]; all of these characteristics were absent in our case. Moreover, CAM is often suspected to be preceded by lymphatic alteration caused by locoregional damage such as that due to axillary dissection, radiation therapy, or trauma [11, 12], which our patient had not experienced.

In this case, differential diagnosis between two diseases was important to provide appropriate management. Occult breast cancer without distant metastasis is usually treated by axillary lymph node dissection with optional management of ipsilateral breast, such as surgery (mastectomy or lumpectomy), whole-breast irradiation, and observation. On the other hand, the best treatment for CAM is still controversial [13, 14]. Although the latest edition of the American Joint Committee on Cancer (AJCC) classified CAM as distant metastasis (M1) [15], there are conflicts over treatment strategies for CAM: systemic treatment without surgical intervention versus locoregional treatment such as surgery and radiotherapy.

In summary, we reported a case of an occult breast cancer presenting with axillary metastasis and synchronous contralateral early-stage breast cancer. Axillary metastasis without clinical evidence of ipsilateral breast cancer is not common, but also not too rare. When such cases are encountered, clinicians should be open to all possible diagnostic options until the final decision is made; they also need to be well informed about the characteristics of each option. Occasionally, surgical management has to be performed before making the final diagnosis, and also additional surgery might be required depending on the postsurgical diagnosis. Therefore, good communication among clinicians, radiologists, and pathologists throughout the whole process is essential, in addition to sufficient explanation to the patients.

## Data Availability

The datasets used and/or analysed during the current study available from the corresponding author on reasonable request.

## Abbreviations

AC-T: Doxorubicin (adriamycin), cyclophosphamide, and paclitaxel
AJCC: American Joint Committee on Cancer
BI-RADS: Breast imaging reporting and data system
CAM: Contralateral axillary metastasis
CT: Computed tomography
ER: Estrogen receptor
GATA3: GATA binding protein 3
GCDFP-15: Gross cystic disease fluid protein-15
H&E: Hematoxylin and eosin
HER2: Human epidermal growth factor receptor 2
MRI: Magnetic resonance imaging
PET-CT: Positron emission tomography-computed tomography
PR: Progesterone receptor
US: Ultrasonography
